# Science of mind and behaviour or allied health profession? Changes in the organisational location of psychology in Australian universities

**DOI:** 10.1080/00049530.2023.2182140

**Published:** 2023-02-23

**Authors:** Nick Haslam, Naomi Baes

**Affiliations:** School of Psychological Sciences, University of Melbourne, Parkville, Australia

**Keywords:** Psychology, organisations, health, universities, inter-disciplinarity, Australia

## Abstract

**Objective:**

The study aimed to characterise the past and current organisational location of psychology in Australian universities.

**Method:**

Contemporary and archived websites of 38 universities were examined to determine whether, in 2005 and 2022, psychology was located within a health-focused organisational structure and functioned as a stand-alone administrative entity.

**Results:**

Most psychology units are currently stand-alone and located within a health-focused structure. Since 2005 they have gravitated into health-focused structures (36.8% to 68.4%) and become less autonomous (84.2% to 63.2%). These trends diverge from the typical arrangement in top North American and UK psychology units.

**Conclusions:**

Australian psychology academics increasingly work in health-focused structures where their discipline is not administratively autonomous. This trend brings opportunities and risks.

## Key Points

**What is already known about this topic:**

1. Australian academic psychology units sit in varied organisational structures.
2. Most early units were located in Arts or Science rather than health faculties.
3.  The clinical and health-related domains of psychology have expanded rapidly.

**What this topic adds:**

1. Most psychology units now sit in health-focused organisational structures.
2. Units have become more health-focused and less autonomous since 2005.
3. These trends are contrary to the norm in leading UK and North American universities.

## Introduction

Research in meta-science has identified psychology as one of seven “hub sciences”, alongside mathematics, physics, chemistry, earth sciences, medicine, and the social sciences (Boyack et al., 2005; Cacioppo, 2007). Based on co-citation patterns, it sits between social science and neuroscience, with weaker connections to humanities, health sciences, and medical specialities (Klavans & Boyack, 2009). This location reflects psychology’s internal complexity, which encompasses the basic science of cognition and the brain, the social science of groups and culture, and the clinical science of health and illness.

Cronbach (1957) recognised psychology’s heterogeneity, describing experimental and correlational psychology as separate disciplines, the former leaning towards natural science, the latter towards social and clinical science. This division persists (Flis & van Eck, 2018), although psychology research has increasingly gravitated towards natural science and away from the humanities (Haslam et al., 2022; Wieczorek et al., 2021).

Where psychology sits in Australian universities is equally complex. The first Australian psychology departments split off from philosophy within Arts faculties (Buchanan, 2012), and the field was historically grouped among the social sciences as a “poor relation” to the natural sciences (Macintyre, 2010). Psychology is now variously located in faculties dedicated to natural science, social science, health and medical science, or arts and humanities, and taught as an accredited sequence in Arts, Science, Business, and Health and Exercise degrees, among others (Bond, 2016). Its location in specific universities may reflect the confluence of many historical, institutional, and financial factors.

It remains unclear how differing organisational arrangements affect psychology research and education at the local level, and how changes in these arrangements influence them nationally. Organisational structures may tend to favour subdisciplines that align with them. In psychology departments sitting within natural science faculties, for example, experimental psychology and cognitive neuroscience might be expected to prosper relative to less well-aligned subdisciplines. Similarly, nationwide trends towards particular organisational arrangements might selectively advantage some subdisciplines.

This possibility is raised by recent restructures in which psychology units have relocated or merged into health-focused faculties (i.e., those focused on understanding and treating physical or mental health conditions, encompassing biomedical science, clinical medicine, public health, and allied health professions). These changes parallel a growing emphasis on psychology education as a pathway into the mental health workforce. Although clinical psychology and other health-related specialities represent just one component of the discipline, this agenda gives them special importance. Relocation of psychology into health-focused organisational structures may boost health-focused subdisciplines at the expense of others.

We hypothesised that the proportion of psychology units (e.g., departments or schools) located in health-focused organisational structures would rise from 2005 (the midpoint of the first decade in which historical data would be readily accessible) to 2022. As a secondary question, we explored whether psychology became less likely to represent a stand-alone administrative unit over this period.

## Method

Our sample comprised the 38 universities offering APAC-accredited psychology programs in 2022. The organisational location of psychology within each university was coded from its website during November 2022 for two timepoints: the present and 2005. Records from 2005 were accessed using the “wayback machine” (https://web.archive.org/), an internet archive that employs web crawler bots to catalogue old websites. For one university (University of the Sunshine Coast) no identifiable psychology-related unit could be found until 2008, so data from that year were used.

For each university and timepoint, we coded two aspects of organisational location. First, we coded whether the psychology unit was located within a health-focused super-ordinate unit (e.g., a Department/School within a health-focused School/Faculty). A unit was coded as “health-focused” if its name explicitly referenced health or medicine or if most of its constituent units were health-related disciplines. Second, we coded whether the psychology unit was administratively stand-alone, rather than merged with other disciplines. It was coded as stand-alone if it was a psychology-only Department/School, and not if it was a part of merged Department/School (e.g., a “discipline” within a combined school of “Psychology and X”). All coding was conducted, checked and agreed by both authors.

## Results

**Figure 1.**
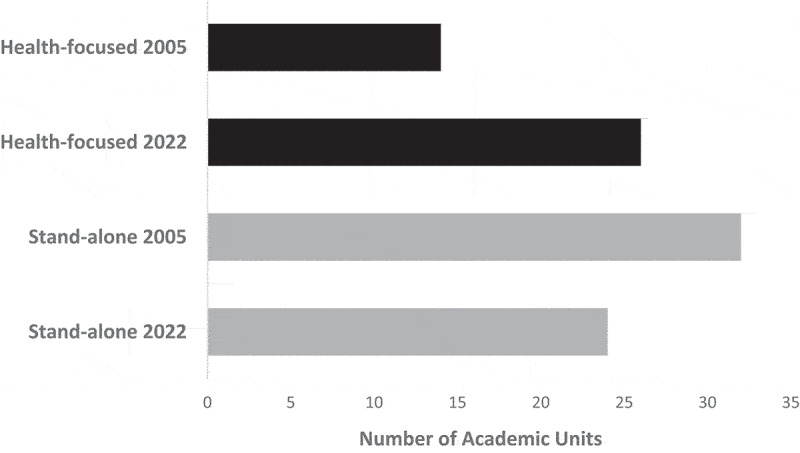
Number of Australian academic psychology units located in health-focused organisational structures, and with stand-alone status, in 2005 and 2022 (N = 38).

Table 1 summarises the coded data, and Figure 1 presents the frequency of each code for 2005 and 2022. It shows that psychology units in 14 universities (36.8%) were located in health-focused organisational structures in 2005 and 26 (68.4%) in 2022. Supporting our hypothesis, McNemar’s test indicates that this increase is significant, χ^2^_(1)_ = 10.29, *p* = .001. Figure 1 also shows that 32 units (84.2%) were stand-alone in 2005 and 24 (63.2%) in 2022, a significant reduction, McNemar’s χ^2^_(1)_ = 4.00, *p* = .045.

**Table 1.** Organisational location of psychology units in Australian universities.

University	Organisational Location		Stand-Alone		Health-Focused		
	2005	2022	2005	2022	2005	2022	
Australian Catholic University	School of Psychology in Faculty of Arts and Sciences	Component of School of Behavioural and Health Sciences in Faculty of Health Sciences	1	0	0		1
Australian National University	School of Psychology in Faculty of Science	Component of School of Medicine and Psychology in College of Health and Medicine	1	0	0		1
Bond University	Department of Psychology in Faculty of Humanities and Social Sciences	Component of School of Social Sciences in Faculty of Society & Design	1	0	0		0
Central Queensland University	School of Psychology & Sociology in Faculty of Arts, Health & Sciences	Component of School of Health, Medical and Applied Sciences	0	0	0		1
Charles Darwin University	Discipline of Psychology in School of Health Sciences	Discipline of Psychology in College of Health & Human Sciences	0	1	1		1
Charles Sturt University	Discipline of Psychology in (1) School of Social Sciences & Liberal Studies and (2) School of Humanities and Social Sciences	School of Psychology in Faculty of Business, Justice and Behavioural Sciences	0	1	0		0
Curtin University	School of Psychology in Division of Health Sciences	Field of Psychology in School of Population Health	1	0	1		1
Deakin University	School of Psychology in Faculty of Health and Behavioural Sciences	School of Psychology in Faculty of Health	1	1	1		1
Edith Cowan University	School of Psychology in Faculty of Community Services, Education, & Social Sciences	Part of discipline cluster in School of Arts and Humanities	1	0	0		0
Federation University Australia	Discipline of Psychology in School of Behavioural and Social Sciences and Humanities	Discipline of Psychology in School of Science, Psychology and Sport	1	1	0		0
Flinders University	School of Psychology in Faculty of Social Sciences	Field of Psychology in College of Education, Psychology and Social Work	1	0	0		0
Griffith University	School of Psychology in Griffith Health group	School of Applied Psychology in Griffith Health group	1	1	1		1
James Cook University	School of Psychology in Faculty of Arts, Education and Social Sciences	Field of Psychology in College of Healthcare Sciences	1	1	0		1
La Trobe University	School of Psychological Sciences in Faculty of Science, Technology and Engineering	Department of Psychology, Counselling and Therapy in School of Psychology and Public Health	1	0	0		1
Macquarie University	Department of Psychology in Division of Linguistics and Psychology	Department of Psychology in Faculty of Medicine, Health and Human Sciences	1	1	0		1
Monash University	Department of Psychology in School of Psychology, Psychiatry and Psychological Medicine	School of Psychological Sciences in Faculty of Medicine, Nursing and Health Sciences	0	1	1		1
Murdoch University	School of Psychology in Division of Health Sciences	Psychology, exercise science, chiropractic, and counselling discipline in College of Science, Health, Engineering and Education	1	0	1		1
Queensland University of Technology	School of Psychology and Counselling in Faculty of Health	School of Psychology and Counselling in Faculty of Health	0	0	1		1
RMIT University	Division of Psychology in School of Health Sciences	Discipline of Psychology in School of Health and Biomedical Sciences	1	0	1		1
Southern Cross University	School of Psychology in Division of Arts	Discipline of Psychology in Faculty of Health	1	1	0		1
Swinburne University of Technology	Area in psychology in Faculty of Life & Social Sciences	Department of Psychological Sciences in School of Health Sciences	1	1	0		1
University of Adelaide	Department of Psychology in Medical School	School of Psychology in Faculty of Health and Medical Sciences	1	1	1		1
University of Canberra	Discipline of Applied Psychology in School of Health Sciences	Discipline of Psychology in School of Health Sciences	1	1	1		1
University of Melbourne	School of Behavioural Sciences in Faculty of Medicine, Dentistry and Health Sciences	School of Psychological Sciences in Faculty of Medicine, Dentistry and Health Sciences	1	1	1		1
University of New England	School of Psychology in Faculty of Arts, Humanities and Social Sciences	School of Psychology in Faculty of Medicine & Health	1	1	0		1
University of New South Wales	School of Psychology in Faculty of Science	School of Psychology in Faculty of Science	1	1	0		0
University of Newcastle	Discipline of Psychology in School of Behavioural Sciences	School of Psychological Sciences in College of Engineering, Science and Environment	1	1	0		0
University of Queensland	School of Psychology in Faculty of Health and Behavioural Sciences	School of Psychology in Faculty of Health and Behavioural Sciences	1	1	1		1
University of South Australia	School of psychology in Division of Education, Arts and Social Sciences	Discipline area in Justice & Society academic unit	1	0	0		0
University of Southern Queensland	Department of Psychology in Faculty of Sciences	Discipline of Psychology in School of Psychology and Wellbeing	1	0	0		1
University of Sydney	School of Psychology in Faculty of Science	School of Psychology in Faculty of Science	1	1	0		0
University of Tasmania	School of Psychology in Faculty of Science, Engineering and Technology	School of Psychological Science in College of Health and Medicine	1	1	0		1
University of Technology Sydney	Discipline of Clinical Psychology in Graduate School of Health	Discipline of Clinical Psychology in Graduate School of Health Disciplines	1	1	1		1
University of the Sunshine Coast	Psychology courses offered in School of Social Sciences^†^	Discipline in School of Health and Behavioural Sciences	0	1	0		1
University of Western Australia	School of Psychology in Faculty of Life and Physical Sciences	School of Psychological Science in the University	1	1	0		0
University of Wollongong	Department of Psychology in Faculty of Health and Behavioural Sciences	School of Psychology in Faculty of the Arts, Social Sciences and Humanities	1	1	1		0
Victoria University	School of Psychology in Faculty of Arts	Part of Clinical Services program in College of Heath & Biomedicine	1	0	0		1
Western Sydney University^‡^	School of Psychology in College of Arts, Education and Social Sciences	School of Psychology in the University	1	1	0		0

^†^= based on 2008 data. ^‡^= University of Western Sydney in 2005. 1 = “Yes”. 0 = “No”.

For comparison, we coded the 2022 organisational location of psychology in the top 10 units in North America and in the United Kingdom, according to the 2023 Times Higher Education psychology subject rankings. Of the 20 universities, 95% had stand-alone psychology units (including one English university with two separate psychology departments) and 15% were located in health-focused structures (0% in North America). By implication, Australian psychology units are less likely to be autonomous and more likely to be aligned with health than world-leading Anglosphere units. The age and prestige of the latter may insulate them against organisational restructures, but as examples of international academic excellence they offer an instructive contrast to contemporary Australian arrangements and trends.

## Discussion

Australian universities’ psychology units have become less administratively autonomous and more likely to sit in a health-focused organisational structure. In 2005, the standard pattern among leading North American and UK universities – a stand-alone psychology unit in a non-health-focused structure – held for most (20; 52.6%) Australian universities. Now it describes just eight (21.1%). The 26 units that sit in health-focused structures vastly outnumber the alternatives: four in science faculties, four in social science, and two in arts and humanities.

Implications of this organisational realignment may be mixed. Health-focused organisational structures may foster applied research, promote interdisciplinary collaborations, and enhance access to health-related research funding. They may afford opportunities to teach students in other disciplines, offer synergies for clinical training, and better support psychology clinics.

There may also be downsides. Publication and citation norms in psychology are generally lower than in clinical, medical and health sciences (Harzing et al., 2014), as is average grant size. Discounting of psychology track records in faculty-level performance evaluations may result, with implications for recognition, reward, and promotion. Psychology units’ strategic plans are required to reflect and implement the strategic objectives of their home faculties, potentially devaluing and discouraging activities that do not align well. For example, research investment may be tilted towards clinical and health psychology research at the expense of basic and social science subdisciplines. Undergraduate psychology teaching can become an attractive source of cross-subsidy for expensive biomedical research on the “cash cow” model, resulting in disproportionate employment of junior teaching-focused staff and shrinking research time. More generally, if psychology functions administratively as an allied health discipline, it may be difficult for it to be a broad, integrative science of mind and behaviour.

The jury is out on whether health- or non-health-focused organisational structures better support academic psychology. Any organisational arrangement is bound to have trade-offs given the heterogeneity of the discipline. However, there are reasons to be vigilant given the creeping transformation we have documented.

The trend away from stand-alone psychology units may also have implications. Whether due to the amalgamation of existing units in search of administrative efficiencies or to the incorporation of new fields, merged units are less likely to represent the disciplinary interests of psychology than autonomous ones. They might also pose challenges around accreditation if psychology does not have autonomous financial responsibility. On the other hand, merged units may cross-pollinate psychology teaching and research in ways that balance any disadvantages. The 12 universities whose units lost stand-alone status between 2005 and 2022 are test cases for evaluating these possibilities.

Our findings confirm trends that interested observers have identified in recent years. The near doubling of universities whose psychology units sit within a health-focused structure is a seismic shift in the organisational company the field keeps. It would be naïve to think this shift will not alter the priorities, incentives, and emphases of Australian academic psychology. Understanding its causes and consequences – for staffing patterns, funding, and research output and impact – is a high priority for the field.

## Data Availability

All data for the study are presented in Table 1 and are also available on request.
